# Experience-dependent glial pruning of synaptic glomeruli during the critical period

**DOI:** 10.1038/s41598-024-59942-3

**Published:** 2024-04-20

**Authors:** Nichalas Nelson, Dominic J. Vita, Kendal Broadie

**Affiliations:** 1 Department of Biological Sciences, Vanderbilt University and Medical Center, Nashville, TN 37235 USA; 2 Department of Cell and Developmental Biology, Vanderbilt University and Medical Center, Nashville, TN 37235 USA; 3 Kennedy Center for Research on Human Development, Vanderbilt University and Medical Center, Nashville, TN 37235 USA; 4 Vanderbilt Brain Institute, Vanderbilt University and Medical Center, Nashville, TN 37235 USA

**Keywords:** Glia, Brain circuit remodeling, JNK signaling, Filamin, *Drosophila*, Cell biology, Neuroscience

## Abstract

Critical periods are temporally-restricted, early-life windows when sensory experience remodels synaptic connectivity to optimize environmental input. In the *Drosophila* juvenile brain, critical period experience drives synapse elimination, which is transiently reversible. Within olfactory sensory neuron (OSN) classes synapsing onto single projection neurons extending to brain learning/memory centers, we find glia mediate experience-dependent pruning of OSN synaptic glomeruli downstream of critical period odorant exposure. We find glial projections infiltrate brain neuropil in response to critical period experience, and use Draper (MEGF10) engulfment receptors to prune synaptic glomeruli. Downstream, we find antagonistic Basket (JNK) and Puckered (DUSP) signaling is required for the experience-dependent translocation of activated Basket into glial nuclei. Dependent on this signaling, we find critical period experience drives expression of the F-actin linking signaling scaffold Cheerio (FLNA), which is absolutely essential for the synaptic glomeruli pruning. We find Cheerio mediates experience-dependent regulation of the glial F-actin cytoskeleton for critical period remodeling. These results define a sequential pathway for experience-dependent brain synaptic glomeruli pruning in a strictly-defined critical period; input experience drives neuropil infiltration of glial projections, Draper/MEGF10 receptors activate a Basket/JNK signaling cascade for transcriptional activation, and Cheerio/FLNA induction regulates the glial actin cytoskeleton to mediate targeted synapse phagocytosis.

## Introduction

Large-scale brain circuit remodeling to refine synaptic connectivity occurs during early critical periods; tight neurodevelopmental intervals when initial sensory experience remodels synapses to reflect environmental input. Critical periods open with the onset of sensory experience and close when circuit stabilization forces resist further change^1–3^. This temporally-restricted circuit remodeling is absolutely essential, as the last chance for significant renovation of genetically-determined brain synaptic connectivity to match the unpredictable, variable environmental input^4^. As in mammals, *Drosophila* critical periods open with sensory experience^5–7^, close to remodeling after a brief window^6,8,9^, and are only transiently reversible during this tightly restricted interval^10–12^. A classic critical period happens in the first few days after *Drosophila* eclosion, when striking brain olfactory circuit antennal lobe synaptic glomeruli remodeling occurs in response to early sensory input^5^. For example, ethyl butyrate (EB)-responsive Or42a receptor olfactory sensory neurons (OSNs) innervating ventral medial 7 (VM7) glomeruli show extensive synaptic pruning in response to EB experience only during this short, defined critical period^10,11^. Importantly, glial cells can function as brain phagocytes to mediate experience-dependent synaptic pruning^13–15^, and glial phagocytosis is the primary mechanism of neuronal remodeling in *Drosophila* injury models and during early *Drosophila* brain development^16–18^.

In *Drosophila* glia, Draper receptors (mammalian MEGF10/Jedi) signal via Basket (mammalian JNK) phosphorylation to drive nuclear translocation for glial transcriptional regulation^19–21^. Puckered phosphatase (mammalian DUSP) dephosphorylates Basket to inhibit this nuclear localization signaling^22–24^. When activated, Basket/JNK translocates into glial nuclei^25^ to cause breakdown of the repression complex that acts to block Activator Protein 1 (AP-1) transcriptional activity^26^. Basket nuclear entry thus increases transcription at the AP-1 motif sites^27,28^. Four of these DNA binding sites regulated by the AP-1 repressor complex are situated directly upstream of the *cheerio* locus^29^. Cheerio is the *Drosophila* homolog of Filamin A (FLNA) that acts as a regulator of actin cytoskeleton dynamics via 1) F-actin cross-linking and 2) a scaffolding function that enables the actin cytoskeleton to respond appropriately to intracellular signaling^30,31^. F-actin remodeling is critical for dynamic extension of glial membrane projections during infiltration, engulfment, and phagocytosis^32,33^. Several *Drosophila* glial classes infiltrate brain synaptic neuropil to act as invading phagocytes in this mechanism^34,35^. We therefore hypothesized that the glial Draper—Basket—Puckered signaling pathway controls Cheerio expression as a key effector of the glial actin cytoskeleton to enable experience-dependent infiltration pruning of the OSN synaptic glomeruli in the *Drosophila* early-life critical period.

In this study, we test EB-responsive Or42a OSN innervation of VM7 glomeruli with critical period EB odorant experience to investigate targeted glial pruning mechanisms. In the *Drosophila* juvenile brain, we find glial membrane projections infiltrate VM7 synaptic glomeruli in response to timed critical period EB experience to mediate dose-dependent pruning. We find Draper receptors essential for experience-dependent synaptic glomeruli pruning, which is completely blocked in *draper* null mutants and with glial-targeted *draper* RNAi. Downstream, we find glial signaling via Basket/JNK and opposing Puckered/DUSP required, with *basket* RNAi and *puckered* overexpression blocking experience-dependent glial pruning. We find critical period odorant experience drives Basket translocation into remodeling glial nuclei, with experience-dependent upregulation of Cheerio expression dependent on glial Draper and Basket signaling. Consequently, we find that glial Cheerio is essential for experience-dependent synaptic glomeruli pruning. Given Cheerio function as an F-actin cross-linking signaling scaffold, we find critical period experience strongly drives experience-dependent restructuring of glial actin cytoskeleton in a circuit-localized mechanism within the target synaptic glomeruli. Taken together, these findings indicate critical period experience triggers Draper receptor to Basket nuclear signaling in glia to induce Cheerio actin cytoskeleton regulation for targeted infiltration pruning.

## Results

### Critical period experience-dependent synaptic glomeruli glial infiltration and pruning

**Figure 1:**
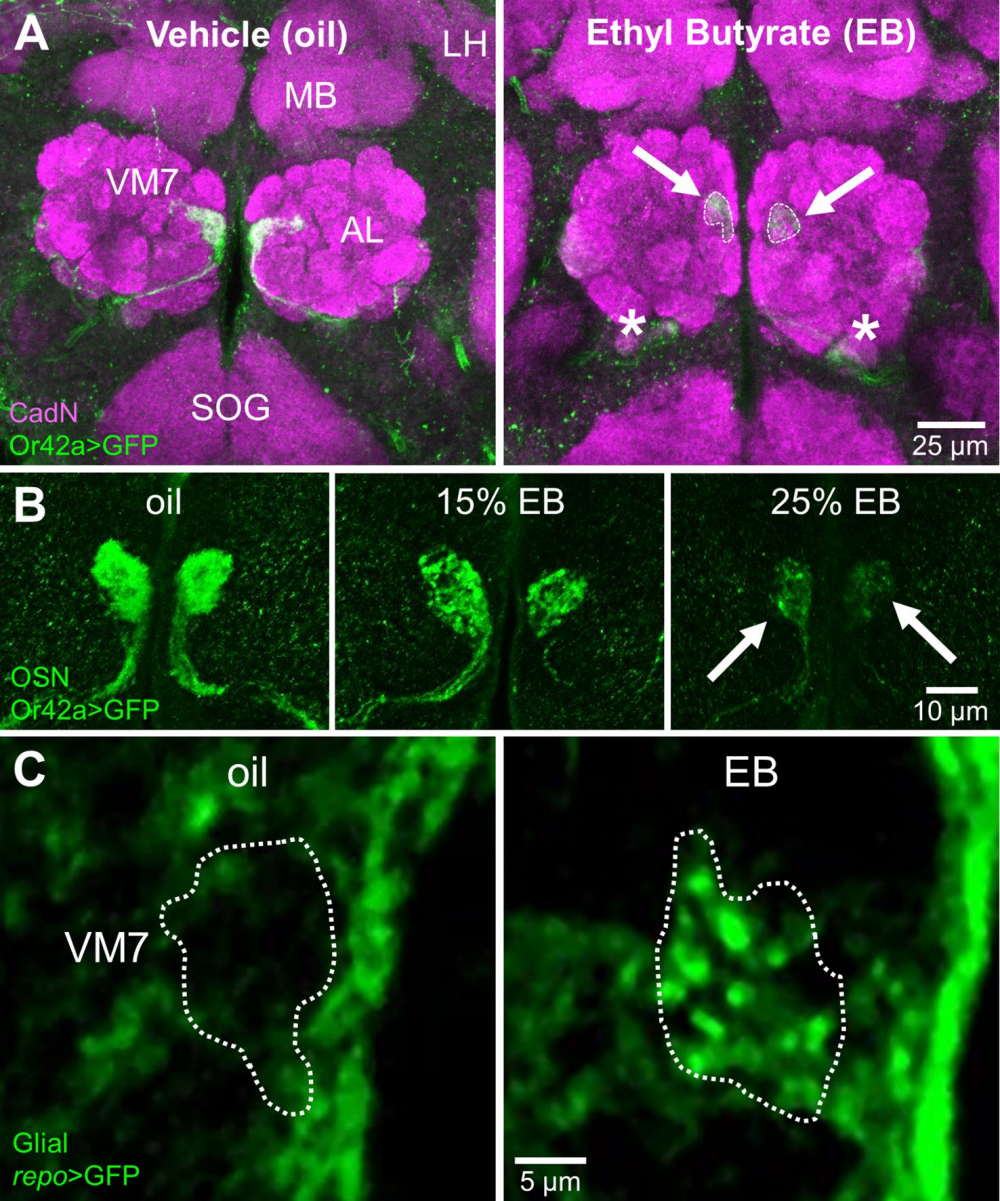
Glial infiltration in experience-dependent synaptic glomeruli pruning. (**A**) Experience-dependent pruning of olfactory sensory neuron (OSN) innervation during the early-life critical period. Low magnification images of central brain antennal lobe (AL) with anti-Cadherin-N (CadN, magenta) synaptic glomeruli labeling and ethyl butyrate (EB) responsive Or42a receptor driving a membrane marker (mCD8::GFP, green) in the Or42a OSNs innervating VM7 glomeruli. Animals exposed to mineral oil vehicle control (left) or EB odorant (right) for 24 h from 0–1 days post-eclosion (dpe). EB experience causes temporally-restricted pruning only in the critical period (arrows), with axon retraction to the AL boundary (asterisks). Abbreviations: antennal lobe (AL), mushroom body (MB), lateral horn (LH), and suboesophageal ganglion (SOG). Scale bar: 25 µm. (**B**) Critical period innervation pruning is dose-dependent. High magnification images of Or42a OSNs innervating the VM7 glomeruli following 24-h critical period exposure to the oil vehicle alone (left), 15% EB (middle), and 25% EB (right), showing the dose-dependent pruning. Scale bar: 10 µm. (**C**) Glial projections infiltrate the EB-responsive VM7 glomerulus in response to 24-h (0–1 dpe) critical period experience. Very high magnification images of glia (*repo*-Gal4 driven UAS-mCD8::GFP, green) following critical period exposure to oil vehicle (left) or 25% EB (right), with experience-dependent infiltration of glial projections specifically into the VM7 glomerulus (dotted outline). Scale bar: 5 µm.

The *Drosophila* brain antennal lobe (AL) provides a well-defined circuit to dissect sensory experience-dependent remodeling of the precisely-mapped olfactory synaptic glomeruli (Fig. 1A). Or42a receptor neurons extend axons targeting the VM7 glomeruli, to synapse onto a single projection neuron (PN)^10^, which projects to mushroom body (MB) learning and lateral horn (LH) innate behavior centers^36,37^. In this study, we use anti-neural cadherin (CadN) to label all synaptic glomeruli^38^ and *Or42a* receptor promoter expression of the mCD8::GFP membrane marker (*Or42a* > GFP) to visualize the specific Or42a OSN innervation of the VM7 glomeruli (Fig. 1A, left). The normal pattern is shown following a 24-h critical period exposure from 0–1 days post-eclosion (0–1 dpe) to the mineral oil vehicle control, which is used to dissolve odorant. Previous work has demonstrated that early-life odorant exposure during this critical period results in dramatic synaptic glomeruli innervation remodeling^5,6,39,40^, with the targeted elimination of Or42a OSN synapses^10,11^. Remodeling is temporally-restricted and transiently-reversible only within the well-defined critical period^10,41,42^. Ethyl butyrate (EB) odorant exposure for 24 h (0–1 dpe) causes strong reduction of Or42a OSN innervation in the VM7 synaptic glomeruli (Fig. 1A, right, arrows). Thus, these neurons exhibit experience-dependent loss of glomeruli innervation in a mechanism completely restricted to the early-life critical period.

The extent of Or42a OSN innervation loss from the VM7 glomeruli is dependent on the level of critical period experience. The odorant vehicle control (mineral oil) reveals the normal pattern of innervation, with dorsally-projecting Or42a OSNs terminating in dense innervation throughout the VM7 glomeruli (Fig. 1B, left). With 15% EB odorant dissolved in the mineral oil, there is a moderate reduction of innervation following 24-h critical period exposure from 0–1 dpe (Fig. 1B, middle). A higher 25% EB concentration results in a more extensive increased loss of the Or42a OSN innervation within the VM7 glomeruli (Fig. 1B, right, arrows). Thus, critical period experience drives dose-dependent loss of synaptic glomeruli innervation. To test glial involvement in this mechanism, we use the glial-specific *repo*-Gal4 driver to express the UAS-mCD8::GFP membrane marker and visualize glial membrane projections (Fig. 1C, *repo* > GFP). In the oil vehicle control, glia surround the AL synaptic neuropil, but only rarely show detectable penetration. Within the target VM7, glia are usually not detectable within the synaptic glomerulus (Fig. 1C, left, white dotted outline). In sharp contrast, 24-h critical period EB exposure (0–1 dpe) causes obvious infiltration of glial projections into the VM7 glomerulus (Fig. 1C, right). These findings suggest glia are infiltrating in a targeted, experience-dependent manner to play a direct role in a critical period pruning mechanism.

### Glial Draper receptors are essential for experience-dependent critical period pruning

**Figure 2:**
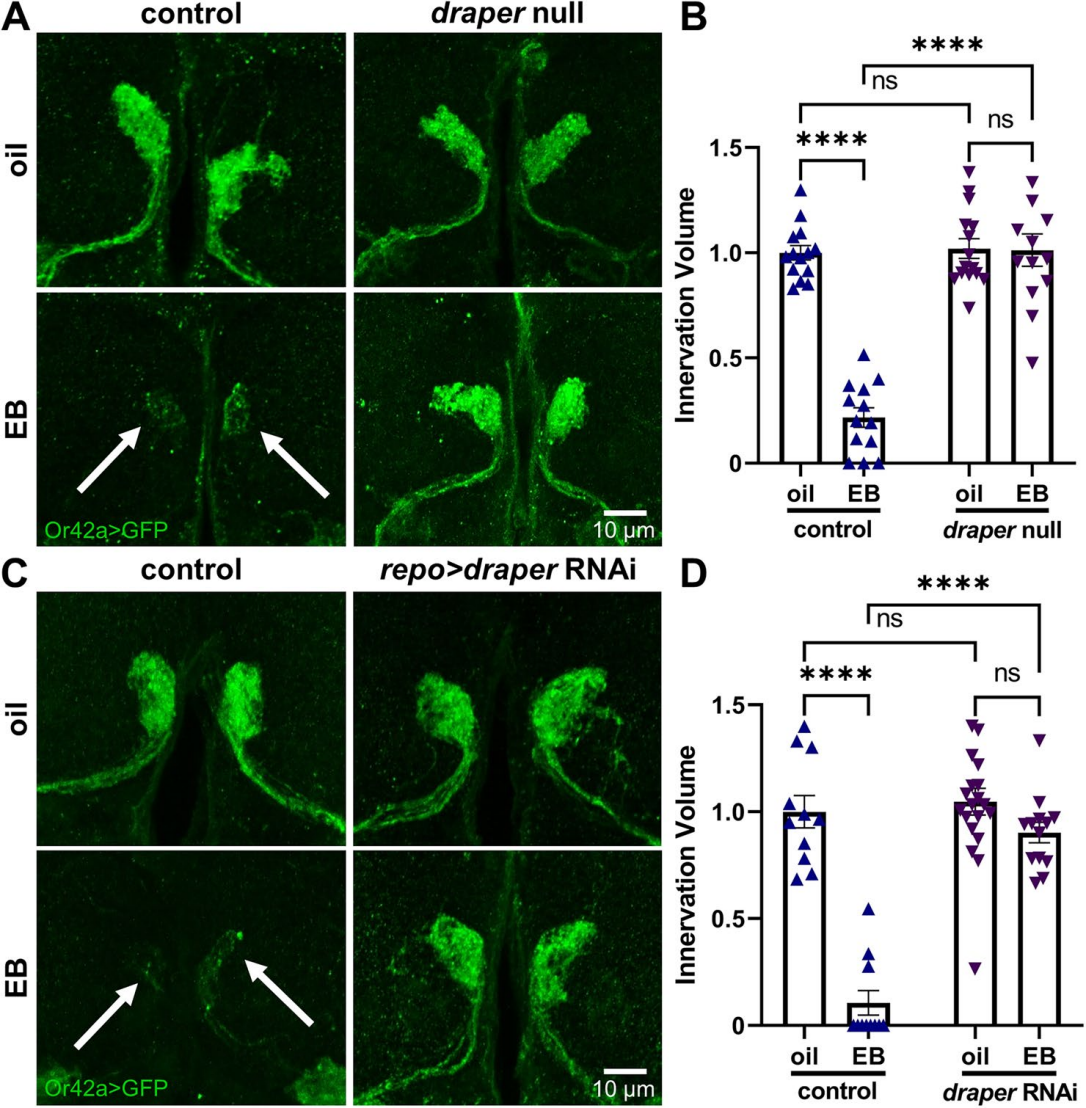
Glial Draper required for experience-dependent critical period pruning. (**A**) Representative images of Or42a OSNs innervating the paired VM7 synaptic glomeruli with *Or42a*-Gal4 driving the membrane marker UAS-mCD8::GFP (*Or42a* > GFP, green) following 24-h critical period exposure to oil vehicle control (top) or 25% EB (bottom). Robust pruning of VM7 innervation occurs in the genetic background control (left, arrows) following 0–1 dpe EB odorant experience, but no pruning happens in the *draper* null mutant (*draper*^*∆5*^, right). Scale bar: 10 µm. (**B**) Quantification of the innervation volume of Or42a OSNs in the VM7 glomeruli in all four genotypes, normalized to the oil vehicle control. Scatterplots show all data points and the mean ± SEM. Significance is indicated as not significant (NS; *p* > 0.05), or significant at *p* ≤ 0.0001 (****). (**C**) Glial-targeted *draper* RNAi prevents critical period experience-dependent pruning. Representative images of Or42a OSNs innervating VM7 synaptic glomeruli in glial attp2 TRiP transgenic driver controls (*repo*-Gal4/attP2; left) or glial *draper* RNAi (right) following 24-h critical period exposure to the oil vehicle (top) or 25% EB odorant (bottom). The robust pruning apparent in the controls (left, arrows) is prevented by glial-targeted *draper* RNAi. Scale bar: 10 µm. (**D**) Quantification of normalized innervation volume of Or42a OSNs in VM7 glomeruli in all four genotypes. Scatterplots show all data points and mean ± SEM. The significance is indicated as not significant (NS; *p* > 0.05), or significant at *p* ≤ 0.0001 (****).

The circuit-localized glial projection infiltration and coincident dose-dependent loss of OSN innervation based on critical period experience led to the hypothesis that glia are mediating synaptic glomeruli pruning. *Drosophila* glial phagocytosis is well characterized following neuronal injury^43–45^, and in early developmental neuronal remodeling^16,46–48^, but it is unknown whether glia act in a critical period pruning mechanism. *Drosophila* Draper (mammalian MEGF10/Jedi) is an engulfment receptor for glial phagocytosis^43,47,49^. To test whether glial phagocytosis is required for critical period pruning, we use both a *draper* null mutant (*draper*^*Δ5*^) and glial-targeted *repo*-Gal4 *draper* RNAi^43^ compared to matched genetic background and transgenic driver controls respectively. As above, *Or42a* receptor driven expression of the mCD8::GFP plasma membrane marker (*Or42a* > GFP) allows the visualization of the Or42a OSN innervation of VM7 glomeruli (Fig. 2A). 24-h critical period exposure from 0–1 dpe is compared between the mineral oil vehicle control (top) and 25% EB odorant dissolved in this mineral oil (bottom). The VM7 glomerular synaptic innervation volume is used to compare the control and experience conditions, within and between genotypes (Fig. 2B). Representative images and quantification for all data points with mean ± SEM are shown for both experience conditions and both *draper* null mutants and glial-targeted *draper* RNAi compared to their controls in Fig. 2.

In the genetic background controls (*w*^*1118*^), the oil vehicle condition shows normal Or42a OSN innervation of the VM7 synaptic glomeruli, whereas the critical period EB experience results in an obvious loss of innervation (Fig. 2A, left, arrows). In contrast, *draper* null mutants (*draper*^*Δ5*^) show a complete blockade of this experience-dependent glial pruning, with the mutants following critical period EB experience indistinguishable from the oil vehicle background control and oil vehicle *draper* null mutant (Fig. 2A, right). Quantification of the Or42a OSN innervation volume normalized to the oil vehicle genetic background control (1.0 ± 0.035 (mean ± SEM), *n* = 14) shows that critical period EB experience causes significant ~ 80% volume reduction (0.217 ± 0.047, *n* = 13; *q*_(51)_ = 14.71, *p* < 1.0 × 10^−14^; Fig. 2B, left). Conversely, there is no difference whatsoever in the Or42a OSN innervation volume in the *draper* null mutant in the oil control condition (1.02 ± 0.047, *n* = 15) compared to the critical period EB experience condition (1.012 ± 0.077, *n* = 13; *q*_(51)_ = 0.1441, *p* = 0.9996; Fig. 2B, right). A two-way ANOVA (2 × 2) comparing innervation volumes reveals significant effects for both genotype (*F*_(1,51)_ = 59.58, *p* = 4.0 × 10^−10^) and odorant exposure (*F*_(1,51)_ = 56.05, *p* = 9.3 × 10^−10^), with a significant interaction between them (*F*_(1,51)_ = 53.93, *p* = 1.6 × 10^−9^; Fig. 2B). These results demonstrate that Draper receptors are required for experience-dependent critical period pruning.

To test a glial-specific role of Draper receptor-mediated phagocytosis, we next use targeted *repo*-Gal4 driven *draper* RNAi. In the driver only control (*repo*-Gal4/attP2), the oil vehicle condition shows normal Or42a OSN innervation of the VM7 synaptic glomeruli, and critical period EB experience causes the expected pruning loss (Fig. 2C, left, arrows). Glial-targeted *draper* RNAi completely blocks this experience-dependent pruning, with the VM7 innervation following EB exposure comparable to both the oil vehicle conditions (Fig. 2C, right). Tukey’s multiple comparison tests normalized to oil vehicle driver control innervation volume (1.0 ± 0.075, *n* = 11) shows ~ 90% pruning with the timed EB exposure (0.105 ± 0.058, *n* = 11; *q*_(51)_ = 12.61, *p* = 2.9 × 10^−11^; Fig. 2D, left). In contrast, glial-specific *draper* RNAi is similar to driver controls with the oil vehicle (1.047 ± 0.062, *n* = 20), and shows no significant pruning of Or42a OSN innervation following 24-h critical period EB experience (0.902 ± 0.049, *n* = 13; *q*_(51)_ = 2.445, *p* = 0.3198; Fig. 2D, right). A two-way ANOVA (2 × 2) comparing innervation volumes shows significant effects for both genotype (*F*_(1,51)_ = 41.68, *p* = 3.9 × 10^−8^) and critical period odor exposure (*F*_(1,51)_ = 63.21, *p* = 1.7 × 10^−10^), with a significant interaction term (*F*_(1,51)_ = 32.87, *p* = 5.3 × 10^−7^; Fig. 2D). Overall, these results clearly demonstrate that glia are essential for experience-dependent pruning in the critical period, in a mechanism requiring Draper receptor function.

**Figure 3:**
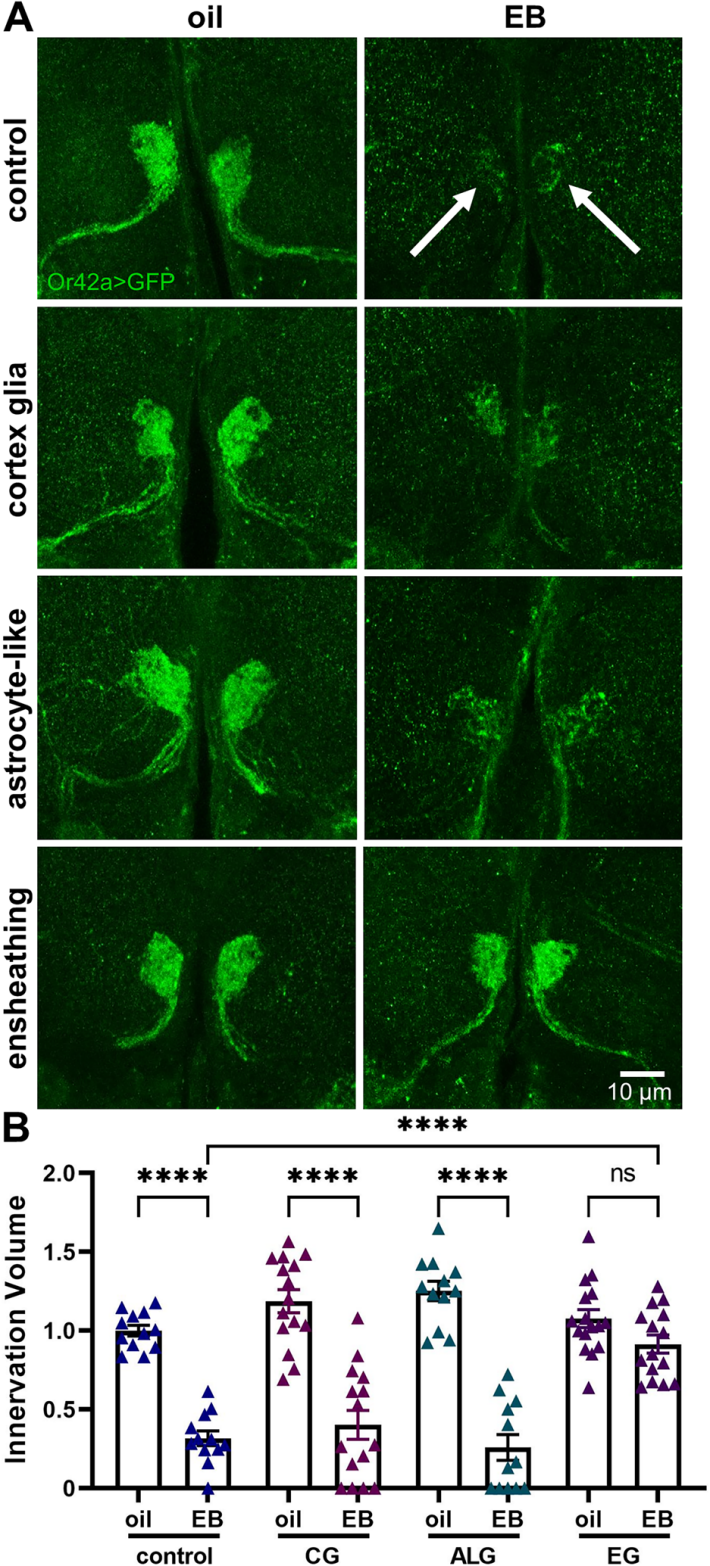
Ensheathing glia mediate experience-dependent critical period pruning. (**A**) Representative images of the Or42a OSN innervation of the VM7 synaptic glomeruli (*Or42a* > GFP, green) with 24-h critical period exposure to oil vehicle (left) or EB (right). Robust innervation pruning occurs in Gal4 driver control (*R56F03*-Gal4/+), with oil vehicle (top left) and following EB experience (top right, arrows). Similar pruning occurs with both cortex glia (*R54H02*-Gal4) and astrocyte-like glia (*R86E01*-Gal4) *draper* RNAi (middle). In contrast, ensheathing glia (*R56F03*-Gal4) *draper* RNAi blocks experience-dependent pruning, with normal innervation in the oil vehicle (bottom left) and indistinguishable maintained innervation following 0–1 dpe EB odorant experience (bottom right). Scale bar: 10 µm. (**B**) Quantification of Or42a OSN innervation volume in VM7 glomeruli in all four genotypes, normalized to the driver oil vehicle control. For each glial class driver, paired 24-h critical period exposure from 0–1 dpe to oil vehicle (left) and 25% EB (right) is shown. Scatterplots show all the data points and mean ± SEM. The significance is indicated as not significant (NS; *p* > 0.05), or significant at *p* ≤ 0.0001 (****).

Cortex, ensheathing, and astrocyte-like glia all function as brain phagocytes^48,50^. We next used glial class-specific drivers to elucidate the phagocytes for synaptic pruning. Oil controls exhibit the normal Or42a OSN innervation, and critical period EB experience causes glial pruning (Fig. 3A, top, arrows). Both cortex (*R54H02*-Gal4) and astrocyte-like glia (*R86E01*-Gal4) *draper* RNAi fails to block experience-dependent synaptic pruning (Fig. 3A, middle images). In sharp contrast, ensheathing glia (*R56F03*-Gal4) *draper* RNAi prevents critical period pruning (Fig. 3A, bottom). Quantification shows Or42a innervation volume normalized to oil vehicle driver control (1.0 ± 0.034, *n* = 12) is pruned by experience (0.318 ± 0.047, *n* = 12; *q*_(101)_ = 9.57, *p* = 2.4 × 10^−8^; Fig. 3B, left). The cortex glia *draper* RNAi oil condition is not significantly different (1.188 ± 0.073, *n* = 15), and is similarly pruned by EB experience (0.402 ± 0.091, *n* = 15; *q*_(101)_ = 12.31, *p* = 1.8 × 10^−12^; Fig. 3B). Likewise, the astrocyte-like glia *draper* RNAi oil (1.252 ± 0.062, *n* = 12) and EB (0.259 ± 0.081, *n* = 12; *q*_(101)_ = 13.93, *p* = 3.3 × 10^−14^; Fig. 3B) conditions show the same degree of innervation pruning. In sharp contrast, ensheathing glia *draper* RNAi prevents experience-dependent pruning, with the oil vehicle innervation (1.076 ± 0.057, *n* = 16) not significantly different following EB experience (0.9148 ± 0.057, *n* = 15; *q*_(101)_ = 2.571, *p* = 0.6097; Fig. 3B, right). These results demonstrate that ensheathing glia are the phagocytes responsible for the experience-dependent synapse pruning during the critical period.

### Antagonistic Basket/Puckered glial signaling drives experience-dependent pruning

**Figure 4:**
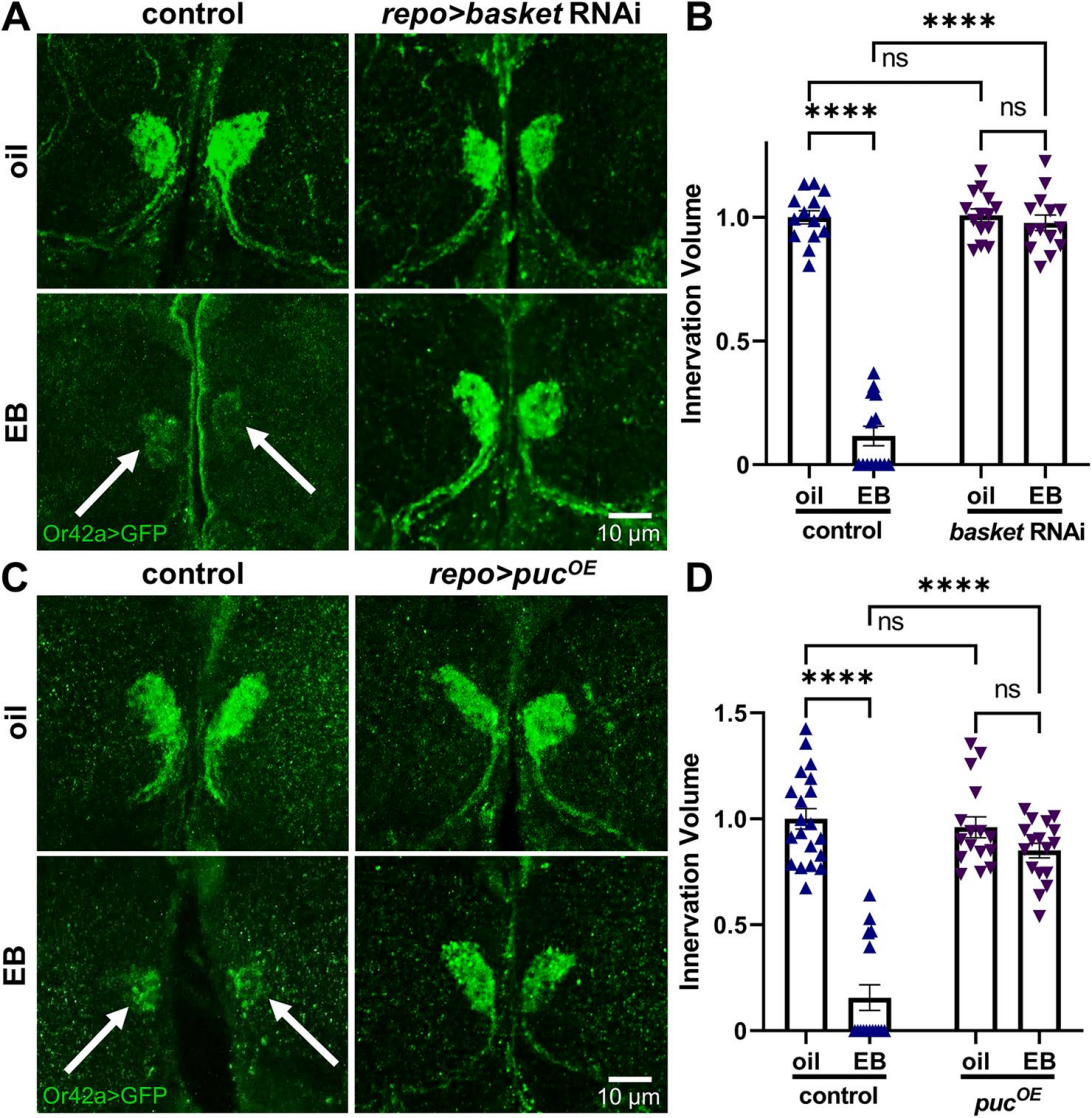
Glial JNK signaling drives experience-dependent critical period pruning. (**A**) Representative images of Or42a OSNs innervating VM7 olfactory synaptic glomeruli (*Or42a* > GFP, green) following 24-h critical period exposure to the oil vehicle control (top) or EB odorant (bottom) from 0–1 dpe. Striking experience-dependent pruning of the VM7 innervation occurs in the glial TRiP driver control (*repo*-Gal4/attP2) following critical period EB exposure (left; arrows), which is completely blocked by glial-targeted *basket* knockdown (*repo*-Gal4 driven *basket* RNAi, right). Scale bar: 10 µm. (**B**) Quantification of Or42a OSN innervation volume in the VM7 glomerulus in all four genotypes, normalized to the oil vehicle control. Scatterplots show all the data points and mean ± SEM. The significance is indicated as not significant (NS; *p* > 0.05), or significant at *p* ≤ 0.0001 (****). (**C**) Glial-targeted *puckered* phosphatase (*puc*) to block *basket*/JNK signaling prevents critical period experience-dependent pruning. The glial driver control (*repo*-Gal4/attP2, left) compared to glial-targeted *puc* overexpression (*puc*^*OE*^, right) with the oil vehicle (top) and EB experience (bottom). Or42a OSN pruning in the VM7 glomeruli (left, arrows) is prevented by glial-targeted *puc*^*OE*^ to block glial *basket*/JNK signaling. Scale bar: 10 µm. (**D**) Normalized quantification of Or42a OSN innervation volume in VM7 glomeruli in all four genotypes. Scatterplots show all data points and the mean ± SEM. The significance is indicated as either not significant (NS; *p* > 0.05), or significant at *p* ≤ 0.0001 (****).

The Draper engulfment receptor activates Basket (Bsk*; Drosophila* JNK homolog) signaling in other phagocytosis mechanisms^21,51–53^, so we tested whether glial Bsk/JNK is required for experience-dependent Or42a OSN pruning within the critical period. Null *basket* mutants are embryonic lethal, so we instead employed glial-targeted *basket* RNAi (*repo*-Gal4 > *basket* RNAi)^54^. The transgenic driver controls (*repo*-Gal4/attP2) exhibit strong glomeruli pruning in response to 24-h EB exposure within the 0–1 dpe critical period (Fig. 4A, left, arrows). In contrast, glial-targeted *basket* RNAi blocks experience-dependent remodeling of the Or42a OSN innervation of the VM7 synaptic glomeruli (Fig. 4A, right). Quantification of innervation volume normalized to oil vehicle driver controls (1.0 ± 0.027, *n* = 14) shows a strong reduction with critical period EB experience (0.116 ± 0.039, *n* = 14; *q*_(52)_ = 28.36, *p* < 1.0 × 10^−15^; Fig. 4B, left). With glial-targeted *basket* RNAi, the oil vehicle condition does not differ significantly from driver controls (1.008 ± 0.026, *n* = 14) and EB experience causes absolutely no pruning (0.9777 ± 0.031, *n* = 14; *q*_(52)_ = 0.969, *p* = 0.9023; Fig. 4B, right). A two-way ANOVA (2 × 2) comparing innervation volumes shows significant effects for both genotype (*F*_(1,52)_ = 194.6, *p* < 1.0 × 10^−15^) and odorant exposure (*F*_(1,52)_ = 215.0, *p* < 1.0 × 10^−15^), with a significant interaction term (*F*_(1,52)_ = 187.6, *p* < 1.0 × 10^−15^; Fig. 4B). Thus, we conclude Basket signaling is essential for experience-dependent pruning.

To further interrogate the role of Basket signaling in critical period glial pruning, we overexpressed the Puckered phosphatase within glia. Puckered (Puc) dephosphorylates Basket to inhibit the signaling cascade^23,24^, providing an independent means to test the glial signaling pathway. The transgenic driver controls (*repo*-Gal4/+) again show strong synaptic glomeruli pruning in response to 24-h critical period EB exposure (Fig. 4C, left, arrows). In contrast, glial-targeted *puc* overexpression (*repo* > *puc*^*OE*^) prevents pruning of Or42a OSN innervation (Fig. 4C, right). Tukey’s multiple comparison tests show Or42a OSN innervation volume normalized to oil vehicle *repo*-Gal4/+ driver controls (1.0 ± 0.048, *n* = 20) is significantly reduced following EB exposure (0.156 ± 0.061, *n* = 16; *q*_(65)_ = 17.52, *p* = 1.5 × 10^−12^; Fig. 4B, left). Conversely, glial-targeted *puc*^*OE*^ causes no change in the oil vehicle treatment condition (0.961 ± 0.049, *n* = 16), and critical period EB experience produces no significant difference in the innervation volume (0.850 ± 0.035, *n* = 17; *q*_(65)_ = 2.206, *p* = 0.4085; Fig. 4D, right). A two-way ANOVA (2 × 2) reveals significant effects for both genotype (*F*_(1,65)_ = 44.43, *p* = 6.7 × 10^−9^) and experience (*F*_(1,65)_ = 94.41, *p* = 2.8 × 10^−14^), with a significant interaction between them (*F*_(1,65)_ = 55.79, *p* = 2.6 × 10^−10^; Fig. 4D). Taken together, these results demonstrate glial Puckered negatively regulates Basket signaling, which is essential for critical period experience-dependent glial pruning.

### Critical period experience drives Basket glial nuclear translocation signaling

**Figure 5:**
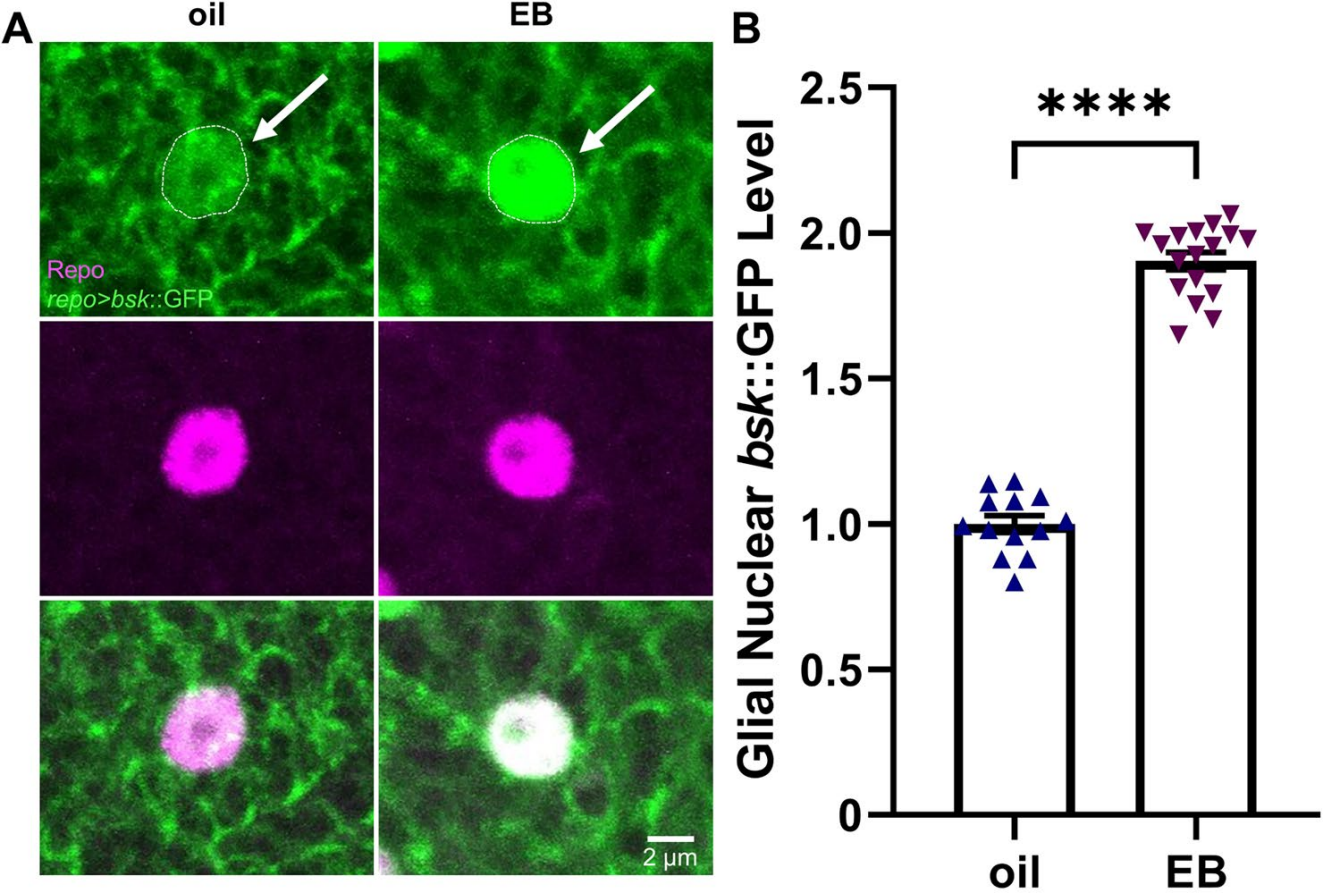
Critical period experience signals Basket/JNK glial nuclear translocation. (**A**) Odorant experience in the critical period drives the nuclear translocation of activated Basket/JNK in glia to modulate downstream transcriptional regulation. Representative high magnification images of glia immediately adjacent to the VM7 glomerulus, which are double-labeled for glial *repo*-Gal4 targeted UAS-*basket*::GFP (*bsk*::GFP, green; top) and anti-Repo to mark the glial nuclei (Repo, magenta; middle), with merged images shown below (white overlap, bottom). 24-h critical period exposure from 0–1 dpe to the oil vehicle alone (left column) and with the EB odorant (right). The signal colocalization in the glial nucleus (white; bottom) indicates *bsk*::GFP nuclear accumulation driven by EB experience in the critical period. Scale bar: 2 μm. (**B**) Quantification of the glial nuclear *bsk::GFP* fluorescence intensity normalized to the oil control, following 24-h critical period exposure from 0–1 dpe to the oil vehicle (left) or 25% EB (right). Scatterplots show all data points and mean ± SEM, with each data point representing the average from the 10 glial nuclei closest to the VM7 glomerulus. Significance is shown at *p* ≤ 0.0001 (****).

Downstream of glial Draper receptor activation, Basket is phosphorylated and thus translocates from the cytosol into the nucleus to drive AP-1-dependent transcription^19,21,53^. In *Drosophila* neural injury models, neurons induce upregulated glial Basket signaling^18^ that results in AP-1 transcriptional activation^21^. Loss of Basket signaling following injury prevents glial phagocytosis^53^, with Basket nuclear translocation and subsequent AP-1 transcriptional regulation required for the glial phagocytosis response. Given the essential Basket role for experience-dependent pruning of Or42a OSNs, we therefore hypothesized that critical period experience should drive a Basket nuclear translocation mechanism to prime glia for synaptic glomeruli remodeling. We use a glial-targeted *basket*::GFP reporter (*repo*-Gal4 > UAS-*bsk*::GFP), together with anti-reversed polarity (Repo) labeling of glial nuclei^55^, to image Basket glial nuclear translocation in animals exposed to oil vehicle only versus 25% EB during the early-life critical period (24 h, 0–1 dpe). The AL is largely devoid of any glial nuclei in all conditions, consistent with the mapping of glial localization in the *Drosophila* brain^44,53^. Thus, the ten closest glial nuclei to the VM7 glomerulus have been imaged, with their *basket*::GFP nuclear fluorescence intensity averaged to represent each single data point. Representative glial nuclei images and quantification with all data points are shown for both critical period experience conditions in Fig. 5.

In animals exposed to the oil vehicle control (24 h, 0–1 dpe), the glial-targeted *basket*::GFP signal is widely dispersed within the cytosol and not detectably enriched in the Repo-labeled glial nuclei compared to the cytosol (Fig. 5A, left, arrow). In contrast, animals with 24-h EB odorant experience in the critical period (0–1 dpe) exhibit strongly elevated levels of *basket*::GFP within glial nuclei, colocalized with the nuclear Repo label (Fig. 5A, right, white). Cytosolic label is still present in animals exposed to critical period experience, however nuclear levels are elevated compared to animals exposed to only oil vehicle (compare glial nuclei; outline, arrows). Importantly, EB experience-dependent *basket*::GFP glial nuclear translocation occurs specifically in glia immediately adjacent to the EB-responsive VM7 glomeruli, indicating circuit-localized glial expression control. Quantification of glial nuclear *basket*::GFP levels normalized to the oil vehicle control (1.0 ± 0.029, *n* = 13; 10 nuclei averaged per data point) shows a highly significant ~ 200% mean nuclear elevation following critical period EB experience (1.904 ± 0.030, *n* = 17; *t*_(28)_ = 21.24, *p* < 1.0 × 10^−15^, two-tailed unpaired *t-*test; Fig. 5B). These results show sensory experience in the early-life critical period results in heightened Basket nuclear translocation within the remodeling glia, which is known to drive glial transcriptional activation enabling the glial phagocytosis of neurons. We therefore next turned to screening for targets of this experience-dependent regulation.

### Experience-dependent Draper-Basket signaling upregulates glial Cheerio expression

**Figure 6:**
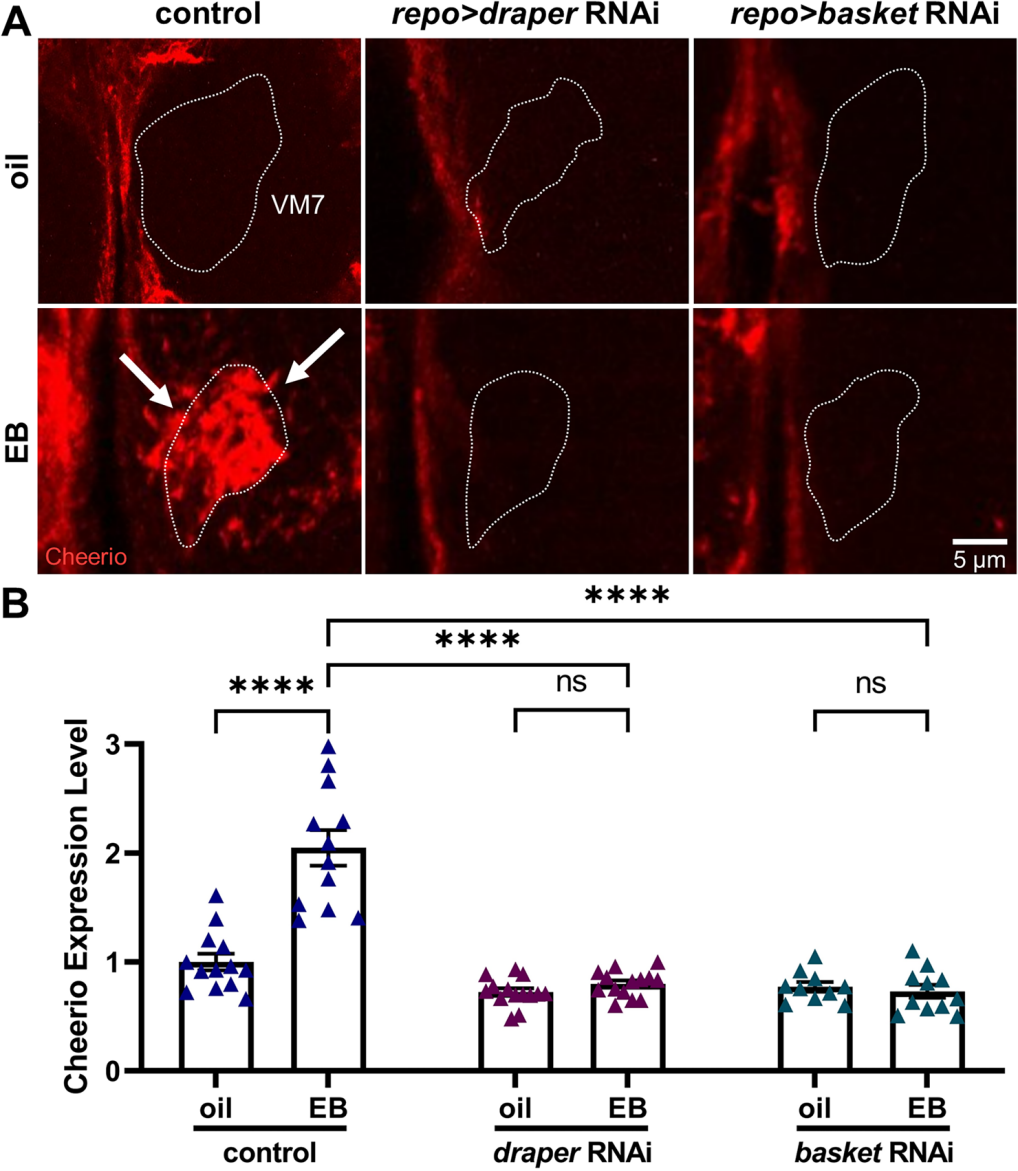
Glial JNK signaling upregulates the F-actin scaffold Cheerio/FLNA. (**A**) Critical period EB experience dramatically upregulates glial Cheerio/FLNA expression downstream of both Draper receptor and Basket signaling in glia. Representative high magnification images of a VM7 glomerulus (dotted outlines) labeled for anti-Cheerio (red) in the glial transgenic driver control (*repo*-Gal4/attP2, left), glial-targeted *draper* RNAi (middle), and glial-targeted *basket* RNAi (right) following the 24-h critical period exposure from 0–1 dpe to the oil vehicle alone (top) or with the EB odorant (bottom). Experience-dependent Cheerio expression upregulation within the VM7 synaptic glomerulus (arrows) completely depends on glial Draper and Basket signaling. Scale bar: 5 μm. (**B**) Normalized quantification of the Cheerio fluorescence intensity expression levels in the *repo*-Gal4/attP2 driver control (left) compared to glial-targeted *draper* RNAi (middle) and *basket* RNAi (right), with both oil vehicle control and EB odorant exposure from 0–1 dpe. Scatterplots show all data points and the mean ± SEM. The significance is indicated as not significant (NS; *p* > 0.05), or significant at *p* ≤ 0.0001 (****).

Upon translocation to the glial nucleus, phosphorylated Basket alleviates a strong repression of AP-1 transcription sites by dissociating an inhibitory histone complex^56–58^. This signaling mechanism thereby mediates the transcriptional activation of AP-1 target genes^59,60^, which encode proteins related to cytoskeleton regulation and cell motility^61,62^. In particular, four AP-1 sites are situated directly upstream of the first untranslated exon of the *cheerio* (*cher*) gene^63^, which encodes the *Drosophila* homolog of the filamentous actin cross-linker signaling scaffold Filamin A (FLNA)^64,65^. Infiltration phagocytosis greatly depends on restructuring of the F-actin cytoskeleton, and Cheerio/FLNA has vital roles in this regulation in both mammal^66,67^ and *Drosophila* cells^29,68^. We therefore hypothesized that critical period experience drives Draper→Basket nuclear translocation signaling to upregulate Cheerio expression within glia, and thus enable the glial actin cytoskeleton rearrangement driving experience-dependent infiltration phagocytosis. We used the very well-characterized anti-Cheerio antibody^29,69^ to test glial Cheerio levels within the VM7 synaptic glomeruli following timed critical period experience. The transgenic driver control (*repo*-Gal4/attP2) was compared to glial-targeted *draper* and *basket* RNAi to test roles of Draper→Basket signaling in experience-dependent Cheerio regulation. Representative images and quantified data for all three genotypes are shown in Fig. 6.

Animals exposed to the oil vehicle control only have antennal lobes almost entirely devoid of detectable Cheerio expression (Fig. 6A, left). While absent within AL synaptic glomeruli, a detectable amount of Cheerio is present within the ensheathing glia (EG) surrounding the neuropil. To assess whether EB experience affects glial Cheerio levels, we focused analyses on the EB-responsive VM7 synaptic glomerulus (Fig. 6A, white dotted outline). Following 24-h critical period EB exposure (0–1 dpe), Cheerio is highly upregulated specifically within the VM7 glomerulus (Fig. 6A, left bottom, arrows). The strongly heightened Cheerio expression is most intense within and directly surrounding the VM7 glomerulus, indicating a circuit-localized critical period experience response. Moreover, the upregulated Cheerio expression replicates the pattern of infiltrating glial membranes driven by critical period EB experience (Fig. 1C), showing elevated Cheerio expression in glial projections. Quantification of anti-Cheerio fluorescence normalized to the oil vehicle control (1.0 ± 0.076, *n* = 13) shows a very significant experience-dependent ~ 200% mean elevation in Cheerio expression with 24-h critical period EB exposure (2.047 ± 0.162, *n* = 12; *q*_(68)_ = 13.21, *p* < 1.0 × 10^−15^; Fig. 6B, left). These results demonstrate a circuit-localized increase in Cheerio expression specifically within the EB-responsive VM7 synaptic glomerulus that is driven by critical period EB experience.

We next tested if Cheerio upregulation is dependent on Draper→Basket signaling by employing glial-targeted RNAi. With animals exposed to oil vehicle only, Cheerio is again absent in the ALs for both *draper* and *basket* RNAi (Fig. 6A, top middle and right). Importantly, the EB experience-dependent upregulation of Cheerio expression is totally blocked by both *draper* and *basket* RNAi (Fig. 6A, bottom middle and right). Compared to oil vehicle controls, Cheerio levels remain unaltered by 25% EB exposure for both of the glial-targeted RNAi lines, showing Cheerio upregulation is downstream of Draper and Basket signaling. Quantification of anti-Cheerio fluorescence levels normalized to the oil vehicle control shows no significant difference with *draper* RNAi (0.7237 ± 0.034, *n* = 14) and no significant increase with EB experience (0.7988 ± 0.032, *n* = 14; *q*_(68)_ = 1.003, *p* = 0.9803; Fig. 6B, middle). Similarly, there is no difference in Cheerio expression in the oil condition for *basket* RNAi (0.7713 ± 0.044, *n* = 10) compared to EB experience (0.7301 ± 0.059, *n* = 11; *q*_(68)_ = 0.4761, *p* = 0.9994; Fig. 6B, right). An ANOVA (3 × 2) to compare the Cheerio expression in all three genotypes in vehicle vs. EB experience shows significant effects for genotype (*F*_(2,68)_ = 61.96, *p* < 1.0 × 10^−15^) and odorant exposure (*F*_(1,68)_ = 30.15, *p* = 6.4 × 10^−7^), as well as the interaction between them (*F*_(2,68)_ = 27.76, *p* = 1.5 × 10^−9^; Fig. 6B). Taken together, these results demonstrate experience-dependent glial Draper→Basket signaling strongly upregulates glial Cheerio expression during the early-life critical period.

### Cheerio remodels the glial F-actin cytoskeleton for experience-dependent pruning

**Figure 7:**
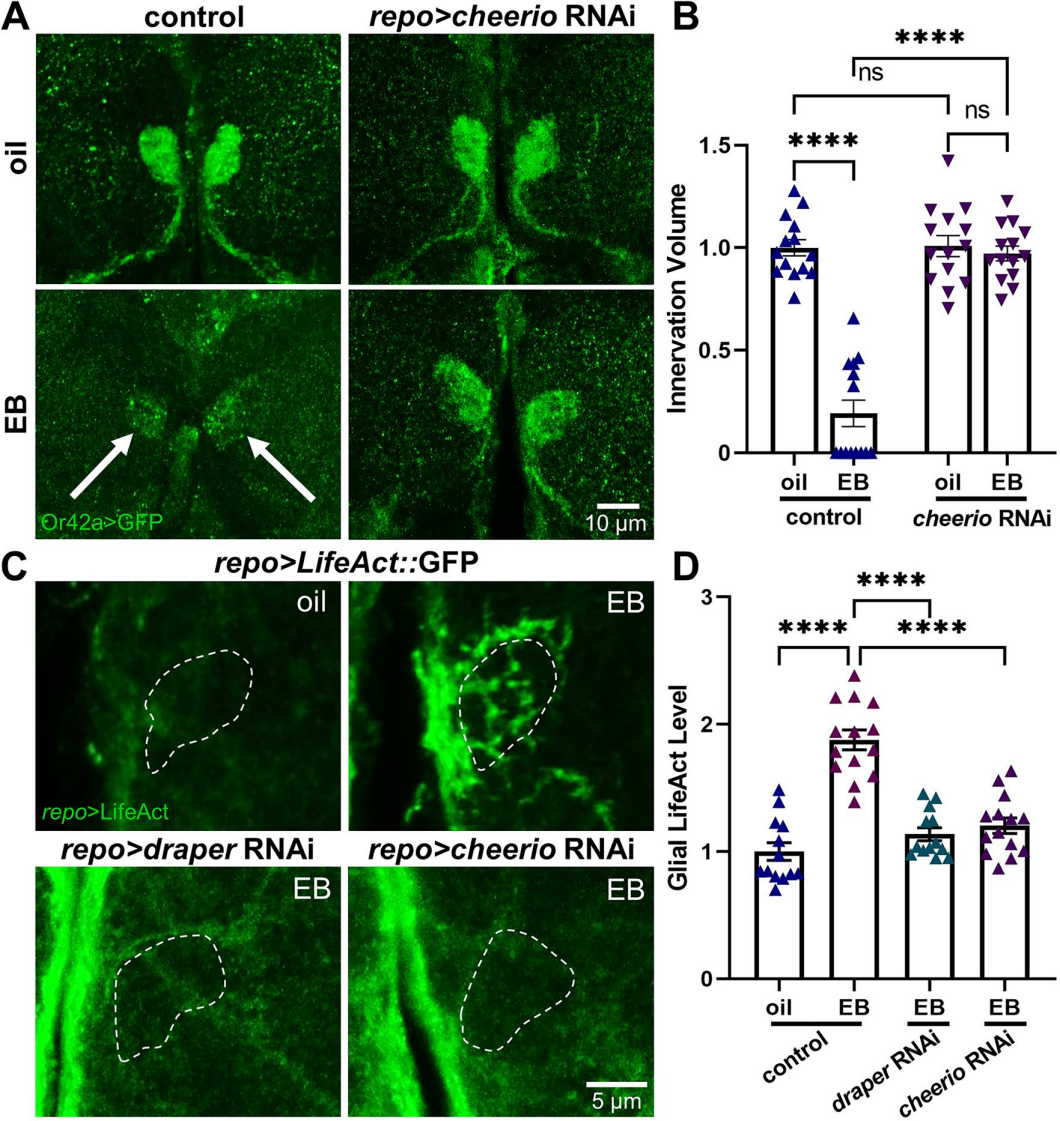
Cheerio remodels F-actin cytoskeleton for experience-dependent pruning. (**A**) Glial Cheerio is required for critical period experience-dependent pruning of olfactory sensory neuron innervation. Representative images of the Or42a OSNs innervating VM7 synaptic glomeruli (*Or42a* > GFP, green) following 24-h critical period exposure to the oil vehicle control (top) or EB odorant (bottom) from 0–1 dpe. Robust glial pruning of the VM7 innervation occurs in the glial transgenic driver control (*repo*-Gal4/attP2) following EB experience (left, arrows), which fails completely with *repo*-Gal4 glial-targeted *cheerio* RNAi (right). Scale bar: 10 μm. (**B**) Quantification of the Or42a OSN innervation volume in the VM7 glomeruli of all four genotypes, normalized to the oil vehicle control. Scatterplots show all data points and mean ± SEM. The significance is indicated as not significant (NS; *p* > 0.05), or significant at *p* ≤ 0.0001 (****). (**C**) High magnification images of glial *repo*-Gal4 driven F-actin marker LifeAct::GFP in VM7 glomeruli (dotted outline) following 24-h critical period exposure to oil vehicle (top left) or EB odorant (top right) from 0–1 dpe, showing the experience-dependent remodeling of the F-actin cytoskeleton. Glial-targeted *draper* and *cheerio* RNAi prevents experience-dependent F-actin induction in VM7 glomeruli (bottom images). Scale bar: 5 μm. (**D**) Normalized quantification of glial LifeAct::GFP fluorescence intensity levels in VM7 glomeruli with 24-h critical period exposure to the oil vehicle or 25% EB. Scatterplots show all data points and mean ± SEM. The significance following a one-way ANOVA is indicated at *p* ≤ 0.0001 (****).

In mammalian glia, disruptions in the ability to regulate the F-actin cytoskeleton result in impaired glial activation and phagocytosis^70,71^. Likewise, *Drosophila* glia require F-actin regulation for the infiltration pruning of MB γ neurons^72^. Thus, F-actin cytoskeletal control plays a vital role in glial phagocytosis function. Given Cheerio/FLNA is an F-actin crosslinker upregulated following early critical period experience, we hypothesized that Cheerio is required for the experience-dependent glial pruning of OSN synaptic glomeruli. To test this hypothesis, we used glial-targeted *cheerio* RNAi^73^ to assess critical period pruning in the EB-responsive VM7 glomerulus. Glial projections infiltrate synaptic neuropil in response to critical period experience (Fig. 1C). Similar to Cheerio/FLNA roles in other cellular contexts^29,30^, we hypothesized Cheerio is required to properly regulate the glial actin cytoskeleton to enable projection infiltration phagocytosis. To test whether the glial F-actin cytoskeleton is restructured in response to critical period experience, we utilized the cell-targeted F-actin biomarker UAS-LifeAct::GFP^74^. Driving this reporter specifically within remodeling glia (*repo*-Gal4 > *LifeAct::GFP*) allows for the visualization of the glial actin cytoskeleton during the critical period. Representative images and quantified results for both the glial-targeted *cheerio* RNAi and LifeAct::GFP F-actin labeling are shown for the oil vehicle controls and critical period EB experience in Fig. 7.

Since critical period experience elevates VM7 circuit-localized Cheerio expression, we first tested glial requirements in experience-dependent Or42a OSN synaptic glomeruli pruning using a glial-targeted *cheerio* RNAi (*repo*-Gal4 > *cheerio* RNAi). Compared to the transgenic driver control (*repo*-Gal4/attP2), which undergoes the expected remodeling, glial-targeted *cheerio* RNAi blocks the pruning mechanism (Fig. 7A). Specifically, glial *cheerio* RNAi prevents experience-dependent pruning of Or42a neurons in response to 24-h (0–1 dpe) critical period EB exposure (Fig. 7A). Tukey’s multiple comparison tests show the normalized innervation volume of the oil vehicle driver control (1.0 ± 0.04, *n* = 14) is significantly reduced by EB experience (0.193 ± 0.064, *n* = 14; *q*_(52)_ = 16.39, *p* < 1.0 × 10^−15^; Fig. 7B, left). However, there is no difference in innervation volume with glial-targeted *cheerio* RNAi in the oil vehicle (1.008 ± 0.052, *n* = 14), and no significant difference in innervation volume following critical period EB experience with glial *cheerio* RNAi (0.971 ± 0.036, *n* = 14; *q*_(52)_ = 0.7425, *p* = 0.9527; Fig. 7B, right). A two-way ANOVA (2 × 2) comparing innervation volumes shows significant effects for both genotype (*F*_(1,52)_ = 63.77, *p* = 1.3 × 10^−10^) and odorant exposure (*F*_(1,52)_ = 73.36, *p* = 1.6 × 10^−11^), with a significant interaction between them (*F*_(1,52)_ = 61.19, *p* = 2.4 × 10^−10^; Fig. 7B). Thus, there is an essential requirement for the glial Cheerio actin cross-linker signaling scaffold in critical period pruning.

Glial projections specifically infiltrate EB-responsive VM7 synaptic glomeruli with critical period EB experience (Fig. 1C), and the Cheerio F-actin cross-linker is absolutely required for pruning (Fig. 7A). We therefore hypothesized that glial F-actin cytoskeleton control in response to critical period odorant experience must be the linking mechanism. To visualize glial actin cytoskeleton changes within the VM7 glomerulus, we employ the F-actin marker LifeAct::GFP driven by *repo*-Gal4 within glia (*repo* > *LifeAct::GFP*, Fig. 7C). In the oil vehicle control, glial-targeted LifeAct is present at low levels around the AL, but is largely undetectable in the VM7 glomerulus (Fig. 7C, top left, white dashed outline). In contrast, 24-h EB exposure during the critical period (0–1 dpe) causes a huge increase in the glial F-actin cytoskeleton around and within the EB-responsive VM7 glomerulus (Fig. 7C, top right). Glial-targeted *draper* and *cheerio* RNAi block experience-dependent induction of F-actin in VM7 (Fig. 7C, bottom). Quantification of the VM7 LifeAct::GFP fluorescence intensity normalized to the oil vehicle control (1.0 ± 0.070, *n* = 13) shows a highly significant experience-dependent increase following critical period EB experience (1.878 ± 0.079, *n* = 14; *q*_(50)_ = 13.19, *p* = 5.0 × 10^−12^; Fig. 7D). Overall, these results show that critical period experience activates glial Draper→Basket nuclear translocation signaling to drive Cheerio expression, and thus regulate the glial actin cytoskeleton to enable experience-dependent glial pruning in the early-life critical period.

## Discussion

We discover an experience-dependent glial pruning mechanism in a critical period of the powerful *Drosophila* genetic system. We find glia are recruited to synaptic glomeruli in response to critical period sensory experience to mediate dose-dependent pruning (Fig. 1). Using a combination of mutants, transgenic RNAi and glial-targeted expression studies, we dissect core mechanisms of critical period pruning. We find the glial Draper engulfment receptor (MEGF10/Jedi) drives experience-dependent pruning (Figs. 2,3). We find downstream signaling antagonism between positive Basket (JNK) and negative Puckered (DUSP) functions controls critical period glial pruning (Fig. 4). We confirm *draper* RNAi with a *draper* null, and *basket* RNAi results with *puckered* phosphatase overexpression. We find early-life sensory odorant experience induces activated Basket translocation into remodeling glia nuclei (Fig. 5), driving *cheerio* gene transcription to strongly upregulate Cheerio (FLNA) expression in the glia infiltrating synaptic glomeruli (Fig. 6). We discover this F-actin linking signaling scaffold is absolutely essential for targeted critical period glial pruning, and consequently that sensory experience drives the remodeling of the F-actin cytoskeleton in glia infiltrating synaptic glomeruli (Fig. 7). Together, these results reveal a glial pruning mechanism that is experience-dependent and temporally-restricted, connecting Draper receptor activation, nuclear translocation signaling, and F-actin cytoskeleton regulation.

Different classes of olfactory sensory neurons can either expand or retract synaptic arbors based on critical period experience^5,6,42^. The Or42a OSNs exhibit striking synapse elimination^10,11^. We discover glia infiltrate synaptic glomeruli in response to critical period experience to mediate dose-dependent pruning (Fig. 1). Only the EB-responsive VM7 glomerulus has been tested so far, and studies are needed for other odorant-selective glomeruli to determine the generalization of this mechanism. Glia subclasses differentially refine OSN synaptic architecture in a Draper-dependent mechanism^15^. Three glial classes function as phagocytes and can act cooperatively for neuronal phagocytosis in *Drosophila* juvenile brains^47,75^. In the critical period, we find only ensheathing glia employ Draper for experience-dependent synapse pruning (Fig. 3). Draper receptors activate Basket/JNK signaling to induce neuronal phagocytosis in early development (larval-pupal transition) and following injury^21,51^. However, there was no link to experience or circuit remodeling. Here, we discover glial Draper→Basket signaling is essential for experience-dependent and temporally-restricted glial pruning (Figs. 2,4). Draper also activates Src42a/Shark signaling^51^, which has not been implicated in this study. The Puckered phosphatase^23,24^ inhibits glial pruning (Fig. 4). Glial-targeted RNAi of the other pathway components (e.g. *hep, jra, kay*)^18,21,53^ could provide additional insights for determining the signaling mechanisms controlling critical period experience-dependent glial pruning.

Draper triggers phosphorylated Basket nuclear translocation for transcriptional activation in glia^18,21,56^. Here, the glial translocation signaling mechanism is imaged using a glial-targeted *basket*::GFP transgenic reporter with the glial nucleus co-labeled using a Repo antibody. We discover critical period sensory experience drives very striking Basket translocation into remodeling glial nuclei (Fig. 5). The glial nuclei remain outside of the synaptic glomeruli^44,76^, and extend infiltrating membrane projections^43,77^ into the neuropil to mediate experience-dependent pruning. We discover circuit-localized signaling around EB-responsive VM7 glomeruli (Fig. 5). Nuclear Basket activates Jun-related antigen (Jra; Jun homolog)/Kayak (Fos homolog) heterodimers^20,63^, which regulate the Activator Protein 1 (AP-1) transcription at target sites, with Jra and Kayak acting in concert^63^. Given homodimers do not replicate heterodimer activity^60^, we would predict that glial-targeted RNAi against either one would reveal a role in critical period glial pruning. Basket signaling acts in both neurons and glia^20,53^, but our results indicate a selective glial requirement in experience-dependent pruning. We could also determine whether Basket signaling has a neuronal function in the critical period mechanism. To our knowledge, we are the first to discover a Basket signaling requirement in the glial pruning of normally-developing brain circuits in *Drosophila,* or conserved signaling in any other model system.

The AP-1 complex binds to four separate promoter sites to regulate transcription of the *cheerio* locus^29^, encoding the Filamin A (FLNA) homolog^69^. Mutant *basket*^DN^ and *kayak* nulls have also been shown to regulate Cheerio/FLNA transcription in a *Drosophila* epithelial tumor disease model^29^. Consistently, we discover both glial-targeted *draper* and *basket* RNAi dramatically reduce glial Cheerio expression within synaptic glomeruli during the critical period (Fig. 6). It is assumed here that the changes in Cheerio levels shown closely reflect AP-1 transcriptional regulation, but direct fluctuations of AP-1 activity could potentially also be tested with TPA-responsive element (TRE) GFP reporters^29^, which might show whether reductions in glial Cheerio expression are caused by reduced AP-1 activity. Note that simultaneous manipulations in separate cell types (neurons and glia) requires dual transgenic systems with separable drivers/responders, which we have not yet been able to achieve in critical period studies. We discover that Cheerio expression is experience-dependent and upregulated specifically in the EB-responsive VM7 synaptic glomeruli dependent on Draper→Basket nuclear signaling (Fig. 6). In addition to the *cheerio* gene, AP-1 also regulates the transcriptional activity at other genetic loci^63^. One pertinent example is AP-1 transcriptional regulation of secreted matrix metalloproteinase 1 (MMP1), which has a proposed role in glial phagocytosis^18^. Future work could test Draper→Basket regulation of MMP1, or even Cheerio and MMP1 both working together, in orchestrating glial pruning functions in the juvenile brain critical period.

Cheerio/FLNA supports microfilaments in orthogonal arrays in dynamic membrane movements^69,78^, cross-linking F-actin filaments, and functioning as a vital intracellular signaling scaffold to control force-generating cytoskeletal motor activities^79,80^. Regulation of the F-actin cytoskeleton is thus central to cell motility^81^ and the complex processes of membrane engulfment and phagocytosis^82,83^. Consistently, glial-targeted *cheerio* RNAi utterly blocks experience-dependent glial pruning during the critical period (Fig. 7). Relatively little is known about F-actin regulation in glia^70,84^ and almost nothing is known about glial actin cytoskeleton regulation within early-life critical periods^85–87^. However, visualizing the glial-targeted F-actin marker LifeAct::GFP^74^, we clearly observe sensory experience-dependent rearrangement of the glial actin cytoskeleton circuit-localized to the EB-responsive VM7 synaptic glomeruli (Fig. 7). Given the absolute requirement of Cheerio for the glial pruning of these connections, glial-targeted *cheerio* RNAi prevents the F-actin cytoskeleton rearrangements in response to experience during the early-life critical period (Fig. 7). Similarly, glial-targeted *draper* RNAi also blocks the experience-dependent regulation of the glial F-actin cytoskeleton. Future work may also reveal that additional actin regulatory proteins, such as the Rho GTPase Rac1^88^, also facilitate this targeted glial pruning mechanism.

Glial phagocytosis of supernumerary synaptic connections during multiple stages of brain development is a central mechanism in the refinement and remodeling of neural circuits^89,90^. EB exposure outside the critical period does not result in significant pruning of the Or42a OSN innervation^10^, showing this glial phagocytosis mechanism is temporally restricted. A large body of work has revealed that mammalian glia utilize a variety of mechanisms to phagocytose and eliminate differentially-active synapses^91,92^, including the MEGF10 receptor for the activity-dependent pruning of retinogeniculate synapses^4,13^. We show here that *Drosophila* glia employ the conserved Draper engulfment receptor to prune central brain olfactory synaptic glomeruli in an early-life, experience-dependent critical period mechanism, highlighting the ever-growing similarity between mammalian and *Drosophila* glial functions^93^. However, very few studies have explored glial phagocytic pruning functions to grossly remodel synaptic connections during critical periods^94,95^, and none, to our knowledge, have done so in the normally-developing *Drosophila* brain. Thus, this study presents an invaluable new model to explore glial pruning mechanisms during a temporally-restricted critical period of heightened brain circuit plasticity, providing a novel forward genetic system to complement the ongoing mammalian model glial studies. Future work will continue to build upon this new *Drosophila* genetic model to elucidate the conserved molecular mechanisms directing glia to infiltrate specific brain neuropils to mediate experience-dependent phagocytosis of targeted synapses. Overall, the work presented here reveals an essential role for glial pruning in the juvenile brain olfactory circuitry during the temporally-transient and experience-dependent critical period.

## Materials and methods

### *Drosophila* genetics

All stocks were reared on standard cornmeal/agar/molasses food at 25 °C. The genetic background control line for the Harvard Transgenic RNAi Project (TRiP) lines is the P{CaryP}attP2 third chromosome insert on the *y[1]v*[*1*] TRiP background. The genetic background control for mutants is *w*^*1118*^, to which all genetic lines are back-crossed^96^. The Bloomington *Drosophila* Stock Center (BDSC; Indiana University, Bloomington, IN, USA) TRiP RNAi lines used are: UAS-*draper* RNAi (BDSC #36732), UAS-*basket* RNAi (BDSC #31323), and UAS-*cheerio* RNAi (BDSC #26307). The *puc* overexpression (OE) line is UAS-*puc*.ORF^97^. The GFP-tagged *basket* line is *w*^*1118*^; UAS-*bsk*::GFP^98^. The GFP-tagged F-actin reporter line is *w*^*1118*^*;* LifeAct::GFP (BDSC #58717)^74^. The transgenic drivers are Or42a OSN-specific *Or42a-*Gal4^99^, pan-glial *repo*-Gal4^100^, and glial class-specific drivers *w*^*1118*^*; R54H02-*Gal4 (cortex glia, BDSC #45784), *w*^*1118*^*; R86E01-*Gal4 (astrocyte-like glia, BDSC #45914), and *w*^*1118*^*; R56F03-*Gal4 (ensheathing glia, BDSC #39157)^34,47,51^.

### Odorant exposure

Critical period ethyl butyrate (EB) odorant exposure was done as previously reported^10,40^. Animals were staged as dark pupae (4 days after puparium formation at 25 °C) into separate vials based on genotype and odor exposure. A fine stainless-steel mesh was placed over the top of each vial and secured with tape to contain the flies but still allow free airflow. The vials were placed into an airtight 3700 ml Glasslock container with either 1 ml of the vehicle only (mineral oil) or EB in mineral oil (Sigma-Aldrich; 15–25% v/v EB) in 1.5 ml microcentrifuge tubes secured in the middle of odorant chambers. The odorant chambers were placed in temperature-controlled, humidified incubators at 23 °C on 12 h light/dark cycles. After 4 h in the incubators, eclosed flies were rapidly transferred to clean tubes with fresh vials. Animals were then kept in the odor chambers for an additional 20 h before being processed for immunocytochemistry.

### Immunocytochemistry

Staged *Drosophila* brains were dissected in phosphate-buffered saline (PBS) at room temperature (RT). Brains were then fixed in 4% paraformaldehyde (PFA) + 4% sucrose in PBS (pH 7.4) with constant circular rotation for 30 min at room temperature (RT). The brains were next washed 3X in PBS. After washing, the brains were placed in blocking solution (1% bovine serum albumin (BSA) + 0.5% normal goat serum (NGS) in PBS + 0.2% Triton-X 100 (PBST)) for 1 h with constant rotation. Brains were incubated at 4 °C overnight with primary antibodies in blocking solution (0.2% BSA, 0.1% NGS in PBST), and then washed 3X in PBST for 20 min with constant rotation. Primary antibodies used: chicken anti-GFP (Abcam, ab13970; 1:1000), rat anti-Cheerio (1:1000)^29^, rabbit anti-Repo (a kind gift from Dr. Benjamin Altenhein, University of Cologne, Germany, 1:1000), and rat anti-CadN (Developmental Studies Hybridoma Bank (DHSB); 1:50). After washing 3X in PBST for 20 min, brains were incubated with secondary antibodies in blocking solution for 2 h at RT, followed by 3X final washes with PBST and PBS for 20 mins^101^. Secondary antibodies used: 488 goat anti-chicken (Invitrogen, A11039; 1:250), 546 goat anti-rat (Invitrogen, A11081; 1:250), and 568 goat anti-rabbit (Invitrogen, A11011; 1:250).

### Confocal imaging

Brains were mounted in Fluoromount-G Mounting Medium (00-4958-02) under a glass coverslip (No. 1.5H, Carl Zeiss). Double-sided tape was used as a spacer between the brain and coverslip, and slides were sealed with clear nail polish (Expressie, Essie). Slides were imaged using a laser-scanning confocal microscope (Carl Zeiss LSM 510 META) at 1024 × 1024 resolution, and then projected using ZEN microscopy software^47^. Low magnification images (Figs. 1, 2, 3, 4, 5, 7A) were taken with a 40× oil objective, and high-magnification images (Figs. 5, 7C) were taken with a 63 × oil objective. Imaging settings were unchanged within all biological replicates in all experiments.

### Image analyses

All images were blinded prior to analyses. For innervation volume measurements, hand-drawn ROIs were created around the maximal borders of the VM7 glomerulus and the FIJI plugin 3D Objects Counter was used to quantify volume (RRID:SCR_002285). All quantification was innervation volume, not intensity, so fluorescence background had no effect. For analyses of *basket*::GFP, ImageJ max intensity projections from 15 to 20 slices were created. The ten (10) glial nuclei closest to the VM7 glomerulus were selected and hand-drawn ROIs were created using their outlines. The mean fluorescence intensity of the *basket*::GFP signal in the 10 ROIs was used to quantify nuclear basket localization for a single data point. For analyses of Cheerio and LifeAct::GFP, an oval 150 × 150 pixel ROI centered on the VM7 glomerulus was created, with the mean ROI fluorescence calculated for the entirety of the glomerulus volume (~ 16–20 slices) to quantify intensity.

### Statistical analyses

All statistical tests were performed using GraphPad Prism software (v9.0). All data sets were analyzed using a ROUT outlier test with Q set to 1%. All data sets were subject to a D’Agostino-Pearson normality test. All normal data sets were analyzed using parametric tests. For normal data within a single genotype, an unpaired two-tailed *t* test was used. For data comparing ≥ 2 genotypes, a two-way ANOVA was used with odorant exposure and genotype as independent variables, followed by Tukey’s multiple comparison tests to analyze both genotypes and treatment conditions. All figures show scatterplots with all data points, as well as the mean ± SEM. Significance is shown as *p* < 0.05 (*), *p* < 0.01 (**), *p* < 0.001 (***), *p* < 0.0001 (****), and *p* > 0.05 indicated as not significant (ns).

## Data Availability

The datasets used and analyzed during the current study are available from the corresponding author on reasonable request.
